# Impact of clinical pharmacist discharge prescription review on the appropriateness of antibiotic therapy: a retrospective comparison

**DOI:** 10.1007/s11096-022-01503-7

**Published:** 2022-11-23

**Authors:** Amy Spigelmyer, Catessa Howard, Ilya Rybakov, Sheena Burwell, Douglas Slain

**Affiliations:** 1 Department of Pharmacy, WVU Medicine - J.W. Ruby Memorial Hospital, Morgantown, WV USA; 2 Department of Clinical Pharmacy, School of Pharmacy, and Section of Infectious Diseases, School of Medicine, West Virginia University, 1124 Health Sciences North, P.O. Box 9520, Morgantown, WV 26506-9520 USA

**Keywords:** Antibiotics, Antimicrobial stewardship, Clinical pharmacists, Hospital discharge, Transitions of care

## Abstract

**Background:**

Inappropriate antibiotic prescribing upon hospital discharge has been identified as a significant problem. Despite high rates of antibiotic prescription errors, there is no widely accepted antimicrobial stewardship initiative to prevent such errors.

**Aim:**

The primary objective of this study was to determine the impact of hospital-based clinical pharmacist discharge prescription review on the appropriateness of antibiotic prescriptions.

**Method:**

This was an observational study comparing the appropriateness of hospital discharge antibiotic prescriptions between two similar internal medicine services. One cohort of patients was admitted to medicine services where rounding clinical pharmacists performed routine antibiotic discharge assessment, and the comparator cohort was admitted to hospitalist services without routine pharmacist discharge antibiotic review.

**Results:**

Our study included 150 cases per cohort. Baseline characteristics were similar between groups, except for increased age (*p* = 0.025) and fewer cases of acute bacterial skin & skin structure infections (*p* = 0.001) in the hospitalist cohort. Antibiotic appropriateness was considerably greater in the medicine (pharmacist) group versus hospitalist group [(83% versus 54%, respectively (*p* < 0.00001)]. The difference in appropriateness was mainly driven by pneumonia and urinary tract infection prescriptions.

**Conclusion:**

Appropriateness of antibiotic prescriptions significantly improved in the setting of pharmacist discharge review. This initiative highlights the important role of clinical pharmacists in outpatient antimicrobial stewardship.

## Impact statements

- Clinical pharmacists can perform meaningful discharge antibiotics assessment to ensure concordance with evidence-based medicine.
- The similarities of the two internal medicine cohort services allow for meaningful comparison of appropriate antibiotic prescribing.
- This study showed that non-infectious diseases clinical pharmacists can have a significant impact on discharge antibiotic prescribing.

## Introduction

While antimicrobial stewardship programs commonly focus on inpatient therapy, over half of human antimicrobial use occurs in the outpatient setting, where stewardship activities are limited [1]. Despite the apparent need for improved stewardship during *transitions of care*, such as upon hospital discharge, many institutions lack effective monitoring of such practices [2]. It has been estimated that 30% to 70% of antibiotic prescriptions at hospital discharge are inappropriate [2–4]. With approximately 250 million antibiotic prescriptions written yearly in the United States, inappropriate antibiotics pose a significant risk to patients and public health [5]. Without intervention, inappropriate antibiotic prescriptions may lead to increased costs, adverse drug reactions, readmission, and antibiotic resistance.

A recent review assessed various interventions to improve antibiotic prescribing upon discharge, including clinician education, pharmacist discharge medication review, antimicrobial stewardship prospective review, order sets, and requirement of indications on prescriptions [6]. While there is no optimal approach to improving the appropriateness of antibiotic discharge prescriptions, the utility of pharmacist discharge prescription review is promising [6–11].

At our hospital, rounding clinical pharmacists routinely provide discharge antibiotic prescription review for patients on inpatient internal medicine services (subsequently referred to as medicine services). As a comparator, our hospital has very similar inpatient hospitalist internal medicine teams (subsequently referred to as hospitalist services) that do not have rounding pharmacists who provide antibiotic discharge prescription review. This subtle difference allows us the ability to simultaneously evaluate any impact of pharmacist discharge prescription review on the appropriateness of antibiotic discharge prescriptions.

### Aim

The primary objective of this study was to determine the impact of hospital-based clinical pharmacist discharge prescription review on the appropriateness of antibiotic prescriptions.

### Ethics approval

The West Virginia University Institutional Review Board approved this project in February 2021 (protocol 2012196414).

## Method

This was a single-center, retrospective cohort study with comparison groups, that included patients who were prescribed antibiotics at hospital discharge for the treatment of pneumonia, urinary tract infections (UTI), *Clostridioides difficile* infections (CDI), acute bacterial skin and skin structure infections (ABSSSI), or Gram-negative bacteremia between January 2019 and July 2020. These diseases were chosen because they were the most common for which antibiotics were prescribed and there were current evidence-based guidelines. The two cohorts included patients on either hospitalist services or medicine services. Both types of service teams are led by internal medicine certified physicians that spend the majority of their time on inpatient care at the hospital. The medicine team physicians also attend to some patients in outpatient clinics. This study was conducted at West Virginia University Hospitals (corporate name is WVU Medicine)—Ruby Memorial Hospital, a 720-bed academic hospital in Morgantown, West Virginia, USA.

Clinical pharmacists review inpatient medication therapy for every patient at our institution. While hospitalist services have remote pharmacist coverage and oversight, these pharmacists do not commonly round (participate in walking care rounds) with the team, and they do not routinely complete discharge medication reviews. Additionally, hospitalist service pharmacists cover numerous services simultaneously. In contrast, medicine services have dedicated clinical pharmacists who cover one medicine service, participate in interdisciplinary rounds and complete discharge medication reviews. None of these pharmacists would be considered “infectious diseases” pharmacists, as they did not complete infectious diseases specialty training. Medicine pharmacists review all discharge medications for their service patients during normal business hours (Monday-Friday, 07:00–15:30).

Study patients were identified by the occurrence of antibiotic discharge prescriptions. Patients were eligible for inclusion if they were ≥ 18 years of age, prescribed oral antibiotics upon discharge for the indications listed above, and were admitted to hospitalist or medicine services. Patients were excluded if they were immunocompromised, pregnant, followed by the outpatient parenteral antimicrobial therapy service, discharged on the weekend, transferred from an outside facility without the ability to clarify previous antibiotic therapy, prescribed prophylactic or suppressive antibiotics, received therapy for concomitant infections requiring extended durations of therapy, or if patients left against medical advice. Immunocompromised was defined as CD4 < 200 cells/mm^3^, absolute neutrophil count < 500 cells/mm^3^, or history of transplant.

The primary outcome was *appropriateness of antibiotic prescriptions*. Appropriateness was retrospectively determined by evidence-based guidelines endorsed by the Infectious Diseases Society of America (IDSA), primary literature, and infectious-diseases (ID) trained pharmacist review. First, appropriateness was independently determined by two ID pharmacists. When the determination of appropriateness differed, a third ID pharmacist assessed appropriateness to determine the result. Secondary outcomes included 30-day readmission rates, 30-day readmission rates due to infectious complications, 30-day CDI rates, and determination of prescribing error types. Prescribing errors included unnecessary antibiotic, dose, frequency, duration, and antimicrobial agent. Patient demographics and clinical characteristics were also collected, including therapeutic indication, infectious diseases consultation, multi-drug resistant organisms (defined as resistant to at least three different classes of antibiotics), and antibiotic prescribing. A sample size calculation identified a minimum of 100 in each arm, to detect a 15% difference with a power of 80% and a significance level of 0.05. Nominal data were compared using the *X* ^2^ or Fisher’s-Exact Test, while continuous data were compared by two-tailed t-tests.

## Results

Of the initial 1,511 patients screened, 300 patients were included in the study, after consideration of inclusion and exclusion criteria. There were 150 patients in each cohort. Baseline characteristics were similar between groups, except for increased age (*p* = 0.025) and fewer cases of ABSSSI (*p* = 0.001) in the hospitalist cohort (Table 1). Pneumonia was the most common indication for antibiotics across both cohorts, comprising 72 (48.0%) patients in the hospitalist cohort and 61 (40.7%) patients in the medicine cohort (*p* = 0.245). A majority of patients received either a penicillin or doxycycline as therapy. The rate of infectious diseases consultation and multi-drug resistance was similar between the two cohorts.

**Table 1:** Baseline demographics and clinical characteristics

Characteristic	Hospitalist patients (No pharmacist review) (*n* = 150)	Medicine patients (Pharmacist review) (*n* = 150)	*p*-value
*Demographic*			
Age (years), median (IQR)	67 (56–76)	59.5 (43.75–75.25)	0.025
Female, n (%)	75 (50)	74 (49)	1.00
Body mass index, median (IQR)	28.9 (23.7–34.4)	29.4 (23.9–34.2)	0.612
CrCl (mL/min), mean	82.89	87.85	0.188
*Comorbidities, n (%)*			
Diabetes	51 (34)	49 (33)	0.809
Heart failure	36 (24)	24 (16)	0.766
Pulmonary disease	8 (5)	11 (7)	0.902
Malignancy	28 (19)	25 (17)	0.112
Hepatic dysfunction	9 (6)	17 (11)	0.637
Renal dysfunction	8	7	0.762
*Intravenous drug use*	7	8	0.15
*Clinical characteristics, n (%)*			
Infectious diseases consult	55 (37)	52 (35)	1.00
Multi-drug resistant infections	29 (19)	26 (17)	1.00
*Therapeutic indication, n (%)*			
UTI	39 (26)	38 (25)	1.00
ABSSSI	12 (8)	33 (22)	0.001
Pneumonia	72 (48)	61 (41)	0.245
Bacteremia	11 (7)	6 (4)	0.318
CDI	10 (7)	11 (7)	1.00
Multiple indications	6 (4)	1 (1)	0.12
*Antimicrobials, n (%)*			
Penicillins	65 (43)	75 (50)	0.298
Cephalosporins	29 (19)	36 (24)	0.401
Doxycycline	54 (36)	47 (31)	0.464
Sulfamethoxazole/trimethoprim	8 (5)	5 (3)	0.572
Fluoroquinolones	22 (15)	18 (12)	0.611
Other antimicrobials	30 (20)	28 (19)	0.884

IQR = Interquartile range. All *P*-values < 0.05 are considered significant. CrCl = Creatinine clearance (Cockcroft-Gault). UTI = urinary tract infection. ABSSSI = acute bacterial skin and skin structure infection. CDI = *Clostridioides difficile* infection

**Fig. 1:**
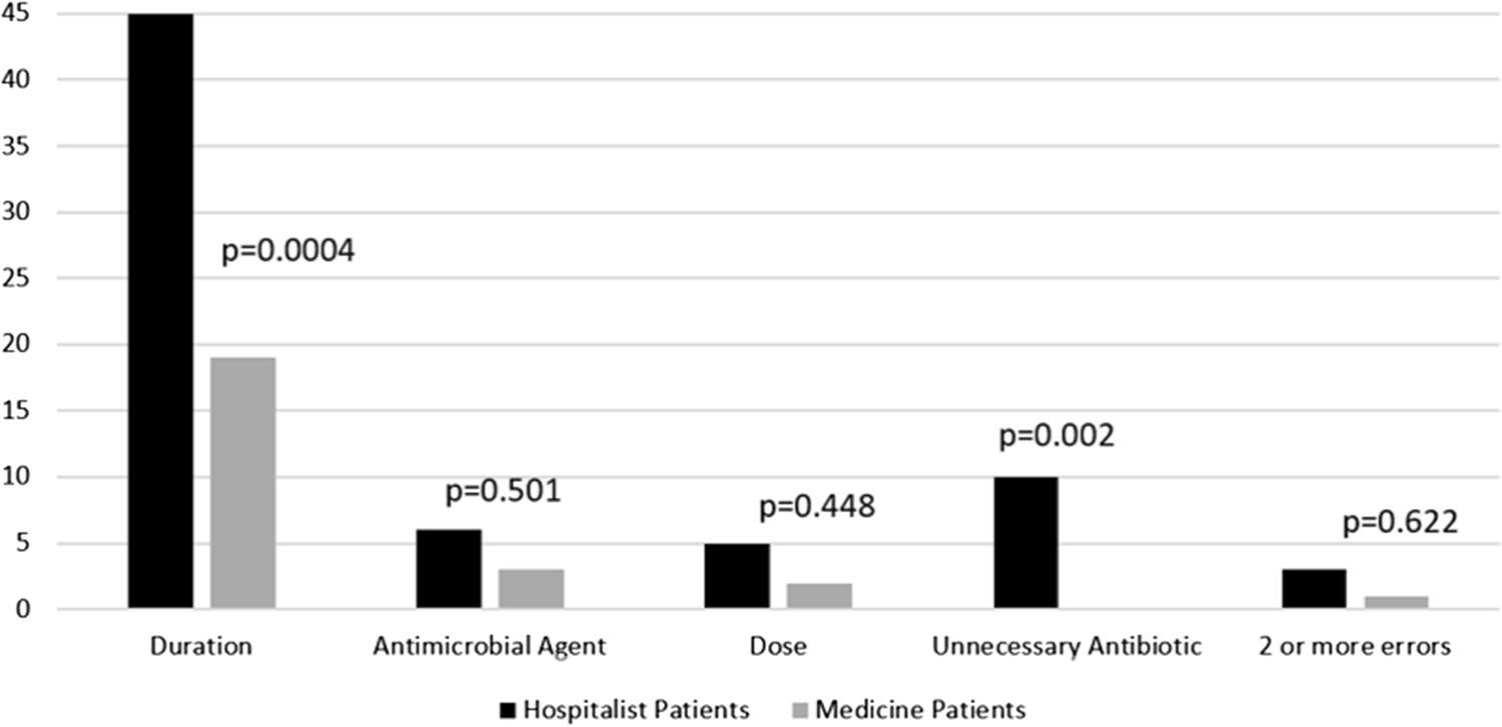
Type of errors in antibiotic discharge prescriptions

Antibiotic appropriateness was significantly higher in the medicine (pharmacist-review) cohort versus the hospitalist cohort, [125/150 (83.3%) versus 81/150 (54.0%), respectively (*p* < 0.00001)]. This difference was driven by increased appropriateness of antibiotic prescriptions for the treatment of UTI (*p* = 0.0019) and pneumonia (*p* = 0.0001) in the medicine cohort versus the hospitalist Cohort (Table 2). The most common type of error (inappropriate order) in antibiotic discharge prescriptions was the duration of therapy, 19 (12.7%) and 49 (32.7%) of inappropriate prescriptions for medicine and hospitalist cohorts, respectively (Fig. 1). The inappropriate trend was typically a longer duration than guidelines recommended. Antibiotic prescriptions were deemed unnecessary in 10 patients on the hospitalist service versus none of the prescriptions on the medicine service (*p* = 0.0017).

**Table 2:** Appropriateness of antibiotic discharge prescriptions by indication

Indications, *n* (%)	Hospitalist [Appropriate Rx] (*n* = 150)	Medicine [Appropriate Rx] (*n* = 150)	*p*-value
UTI	18 (46)	31 (82)	0.0019
ABSSSI	10 (83)	29 (88)	0.65
Pneumonia	42 (58)	54 (89)	0.0001
Bacteremia	3 (27)	0 (0)	0.515
CDI	6 (60)	10 (91)	0.149
Combination	2 (33)	0 (0)	1.00

Rx = Prescription. All *P*-values < 0.05 are considered significant. UTI = urinary tract infection. ABSSSI = acute bacterial skin and skin structure infection. CDI = *Clostridioides difficile* infection. Combination = two of the studied infections

Secondary outcomes did not appear to be different between the cohorts (Table 3). There was a non-statistically significant reduction in 30-day readmission in the medicine cohort [16 of 150 (11%) versus 26 of 150 (17%), *p* = 0.134]. No patients in either cohort had a documented CDI within 30 days of discharge.

**Table 3:** Secondary outcomes

Outcome	Hospitalist patients (*n* = 150)	Medicine patients (*n* = 150)	*P*-value
30-Day readmission, *n* (%)	26 (17)	16 (11)	0.134
30-Day readmission due to infectious complications, *n* (%)	15 (10)	7 (5)	0.119
CDI within 30 days of discharge, *n* (%)	0 (0)	0 (0)	1.000

All *P*-values < 0.05 are considered significant. CDI = *Clostridioides difficile* infection

## Discussion

Expanding antibiotic stewardship initiatives to *transitions of care* and outpatient therapy is imperative for overall patient care. This study found that appropriateness of antibiotic discharge prescriptions significantly increased on medicine services with dedicated pharmacists providing discharge antibiotic prescription review. The results of this study add to the body of literature regarding discharge prescriptions and outpatient antimicrobial stewardship and highlight the importance of clinical pharmacist involvement in improving patient outcomes. Additionally, it is important to note that this study identified an improvement in antibiotic appropriateness without the involvement of an antimicrobial stewardship team. This study shows that non-infectious diseases pharmacists, such as general medicine pharmacists in this study, can drastically improve antibiotic discharge prescription appropriateness.

The specific results in the hospitalist (no pharmacist review) cohort were similar to the findings of previous studies, in terms of rate of antibiotic prescription inappropriateness and the high rates of prescription errors due to duration of therapy [2–4]. A recent multi-hospital cohort study found that 49.1% of UTI and pneumonia prescriptions were inappropriate, with the most common errors being unnecessary antibiotics and duration of therapy. Additionally, while that study found a high rate of antibiotic discharge prescription inappropriateness, there was no association with mortality, readmission, or adverse drug effects [2]. Similarly, a retrospective cohort study found that 53% (79/150) of antibiotic discharge prescriptions were inappropriate, with the most common error being the duration of therapy [4]. Our findings suggest that despite institutional and national guidelines, the treatment of UTI and pneumonia, particularly the determination of duration of therapy, remains a continued area in need of intervention. It is notable that for most patients with “extended durations” of therapy, services prescribed durations of antibiotics without accounting for the days of antibiotic therapy administered during the hospitalization.

This study has several limitations. First, this was a retrospective, single-center study. Therefore, we were unable to confirm that every antibiotic discharge prescription in the medicine cohort was reviewed by a pharmacist, even though this is a routine expectation. Thus, the results of the study may have been impacted by other variables, such as the differences in interdisciplinary rounding between the two cohorts. Second, this study identified patients for inclusion based on antibiotic discharge prescriptions. Therefore, this study did not include patients who were mistakenly discharged without antibiotic prescriptions. Additionally, there was a risk for bias, as the secondary adjudicating ID pharmacists were unblinded to the patient cohort when assessing appropriateness. Lastly, this study utilized a more recent study to support evidence-based guidance on Gram-negative bacteremia [12]. While that article was previously published, providers may not have been aware of institutional guidelines supporting shorter durations of therapy. Therefore, the rates of inappropriate antibiotic prescriptions for the treatment of Gram-negative bacteremia may be falsely elevated in both cohorts.

## Conclusion

Appropriateness of antibiotic prescriptions significantly improved in the setting of pharmacist discharge prescription review. This initiative emphasizes the importance of improved transitions of care and outpatient antimicrobial stewardship. Further prospective studies are needed to determine the full impact of pharmacist discharge prescription review on antibiotic prescriptions.
